# Rapid establishment of KRAS-driven bladder cancer initiation and immune escape models using genetically engineered mice and organoid approaches

**DOI:** 10.3389/fimmu.2026.1726443

**Published:** 2026-03-23

**Authors:** Guoliang Yang, Yishu Wang, Zhangzhengyi Fan, Guojiang Wei, Xuqing Shen, HeJian Zhang, Mengyao Liu, Bin Yu

**Affiliations:** 1 Department of Urology Renji Hospital, Shanghai Jiao Tong University School of Medicine, Shanghai, China; 2 Department of Neurology, Renji Hospital, School of Medicine, Shanghai Jiao Tong University, Shanghai, China; 3 Department of Radiation Oncology, Zhejiang Cancer Hospital, Hangzhou Institute of Medicine (HIM), Chinese Academy of Sciences, Hangzhou, China; 4 State Key Laboratory of Systems Medicine for Cancer, Renji Hospital, School of Medicine, Shanghai Jiaotong University, Shanghai, China; 5 Shanghai Key Laboratory for Cancer Systems Regulation and Clinical Translation (CSRCT), Shanghai, China

**Keywords:** bladder cancer, genetically engineered mouse model (GEMM), KRAS oncogene, organoid culture, tumor immune escape

## Abstract

**Introduction:**

Bladder cancer is the tenth most common cancer worldwide and the sixth most common among men. However, research into representative tumor models for bladder cancer remains underdeveloped, limiting insights into tumor biology and drug development.

**Methods:**

We developed an integrated approach combining a novel gene expression mouse model (GEMM) with advanced organoid technology. This system was tracked longitudinally using single-cell sequencing to monitor tumor evolution and cellular dynamics.

**Results:**

Our model accurately recapitulates the single-cell molecular features and cellular communication networks observed in human bladder cancer. It provides a scalable and physiologically relevant platform for preclinical drug screening.

**Discussion:**

This integrated framework offers a new platform for studying tumor origin and evolution, overcoming key limitations of conventional systems and advancing bladder cancer research.

## Introduction

Bladder cancer is the 10th most common cancer worldwide and the 6th most prevalent in men (1, 2). It is clinically categorized into non-muscle-invasive (NMIBC) and muscle-invasive (MIBC) disease. Although NMIBC generally has a favorable prognosis, 60-70% of cases recur, and 10-20% progress to MIBC, which is associated with poorer outcomes (3, 4). The genomic landscape of bladder cancer is marked by significant chromosomal instability and frequent alterations in tumor suppressor genes such as TP53, PTEN, and RB1 (5–7). In addition to these common alterations, alterations are also observed in RAS oncogenes (HRAS, KRAS, NRAS), which occur in approximately 13% of bladder tumors irrespective of stage or grade (8, 9). Among these, the mutation frequency of KRAS is comparatively low (10). Despite its relatively low frequency, KRAS mutations drive tumor development and progression primarily through the MAPK pathway, which is frequently dysregulated in bladder cancer and promotes tumor cell proliferation, invasion, and survival (11–14). Although the KRAS inhibitor sotorasib has been approved for KRAS-mutated non-small cell lung cancer, its efficacy in bladder cancer remains unproven (15, 16). Consequently, developing novel and effective targeted therapies for patients with KRAS-mutant bladder cancer represents a significant unmet clinical need.

Bladder cancer research relies on several preclinical models, including carcinogen-induced mouse models, genetically engineered mouse (GEMMs) models, patient-derived xenografts (PDXs), and organoid cultures (17). Carcinogen-induced models, such as those generated with N-butyl-N-(4-hydroxybutyl) nitrosamine (BBN), can mimic bladder tumor initiation and progression but show high heterogeneity and limited genetic precision (18–20). GEMMs models allow targeted interrogation of specific genes, but remain constrained by difficulties in inducing invasive phenotypes and in restricting genetic alterations to defined urothelial cells (19). PDXs and organoids preserve key molecular features and are valuable for therapeutic testing, yet their reliance on immunodeficient hosts and variable establishment efficiency restrict their ability to model tumor-immune interactions and disease heterogeneity (21, 22), Overall, although these models have substantially advanced the field, they remain inadequate for faithfully reproducing specific genetic alterations or fully elucidating the mechanisms of tumor initiation.

Here, we developed an alternative approach that integrates GEMMs with organoid technology. Normal urothelial organoids were established, oncogenically transformed *in vitro*, and then transplanted into immunocompetent C57BL/6 mice to generate a bladder cancer model capable of recapitulating immune evasion. Single-cell sequencing was applied throughout the transformation process to delineate dynamic cellular trajectories of tumor initiation and progression. This model not only addresses the limitations of conventional systems in genetic specificity and immune interactions but also provides a novel framework for investigating the origins and evolutionary dynamics of bladder cancer.

## Result

### Establishment of an *in vitro* oncogenic transformation model via GEMM-derived organoids with defined mutations in Kras, Pten, and Trp53

Analysis of the TCGA-BLCA cohort revealed a heterogeneous somatic mutation landscape. Among 414 samples, TP53 was the most frequently mutated gene, with mutations detected in approximately 49% of bladder cancer samples, highlighting its central role in bladder tumorigenesis(**Supplementary Figure S1A**). However, KRAS (~3%, n = 15) and PTEN (~3%, n = 16) mutations were observed at lower frequencies but were consistently present in a subset of patients(**Supplementary Figure 1B–D**). Co-mutation analysis demonstrated that KRAS and PTEN mutations occur both independently and in combination with TP53 alterations, suggesting diverse molecular subtypes within bladder cancer(**Supplementary Figures S1B–D**). Ti/Tv analysis showed characteristic nucleotide substitution patterns consistent with somatic mutational processes in urothelial carcinoma(**Supplementary Figures S1E, F**). Therefore, studying cancer models involving KRAS, PTEN, and TP53 is of significant clinical relevance for understanding the pathogenesis and progression of bladder cancer.

**Figure 1:**
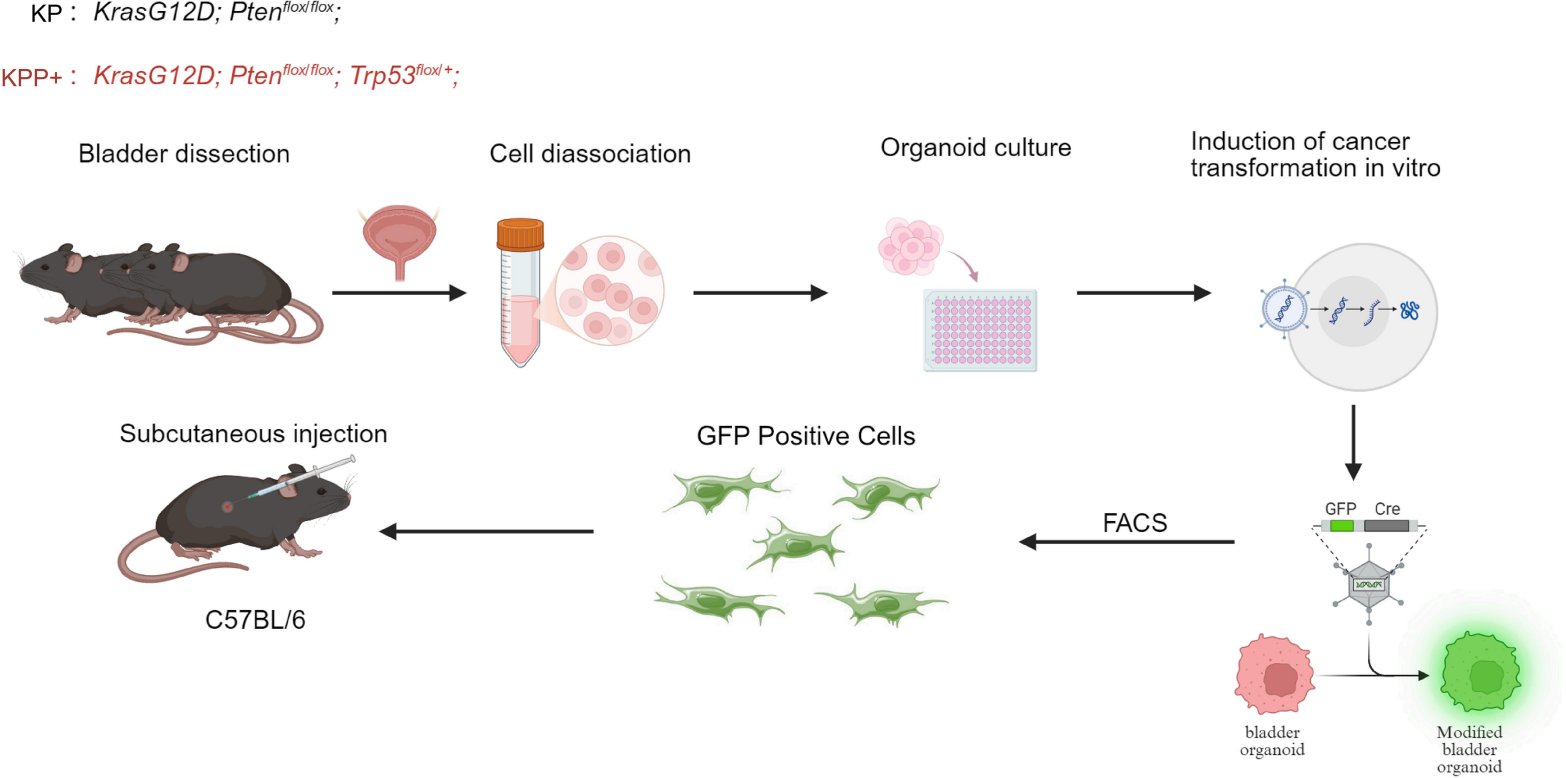
Schematic diagram illustrating the establishment of bladder cancer organoids and the subcutaneous allograft model. Bladders were dissected from genetically engineered mice (Genotypes: KP: *Kras*G12D; *Pten^flox/flox^*; or KPP+: *Kras*G12D; *Pten^flox/flox^*; *Trp53^flox/+^*). The tissues were dissociated into single cells and cultured as organoids. To induce cancer transformation *in vitro*, the bladder organoids were infected with a viral vector expressing GFP and Cre recombinase. Modified GFP-positive cells were subsequently enriched via Fluorescence-Activated Cell Sorting (FACS) and subcutaneously injected into wild-type C57BL/6 mice for *in vivo* tumorigenesis assays.

Because *in vivo* induction of cancer does not allow for the step-by-step and clear documentation of the stages of carcinogenesis, we opted to utilize organoid technology in combination with *Lsp-KrasG12D;Pten^flox/flox^(KP)* & *Lsp-KrasG12D;Pten^flox/flox^;Trp53^flox/+^(KPP+)* GEMMs. This involves establishing normal bladder organoids, which are then infected with a lentivirus carrying Cre and a fluorescent reporter (CoGFP). This infection enables the activation of KrasG12D and deletion of Pten or Trp53—alterations that are common in bladder cancer—thereby generating transformed cancerous organoids. Subcutaneous transplantation of the two tumor organoid types into immunocompetent C57BL/6 mice revealed that only organoids with the *Lsp-KrasG12D;Ptenflox/flox;Trp53^flox/+^* genotype formed tumors, unlike those with only Kras and Pten mutations. These findings demonstrate that the *Pten/KrasG12D* genotype alone is not sufficient for immune evasion and tumor formation. Heterozygous loss of *Trp53* significantly promotes tumorigenic potentia. To delineate the differential impact of KP versus KPP+ genotypes on the processes of tumor initiation and evolution, we employed scRNA-seq to profile normal tissues, organoids (both normal and transformed), and KPP+ tumors at serial time points. This strategy allows us to map the cellular origins of cancer and the genotype-specific pathways governing development and immune evasion(**Figure 1**).

**Figure 2:**
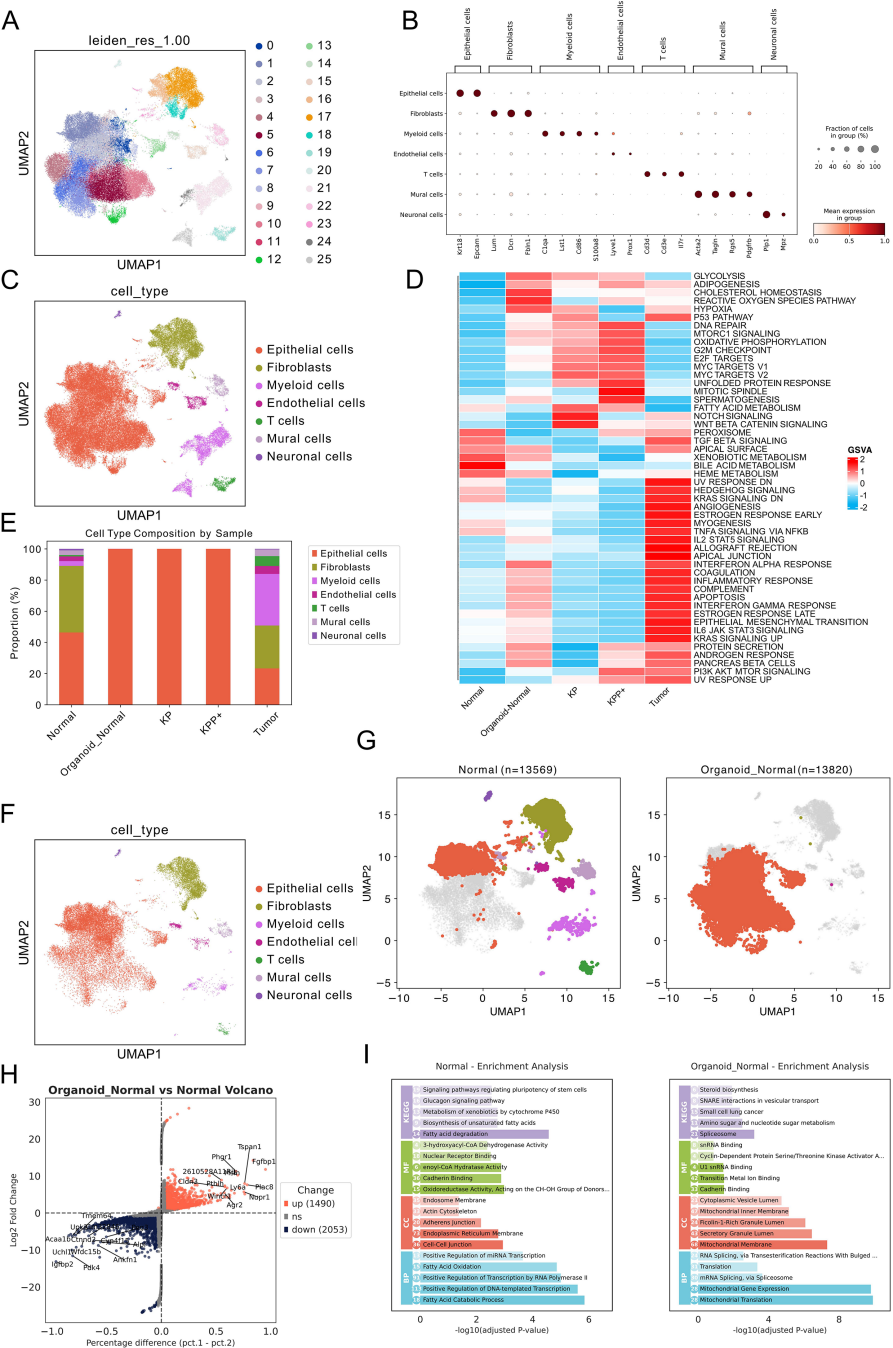
Overview of single-cell RNA sequencing annotation and comparison between normal tissue and normal organoids. **(A)** UMAP clustering of all cells at a resolution of 1.0. **(B)** Dot plot showing cell-specific marker genes following annotation of different cell subpopulations. **(C)** UMAP visualization after completion of cell annotation. **(D)** GSVA differential expression heatmap based on annotated cell subpopulations using the HALLMARK gene set. **(E)** Proportion of different cell subpopulations across all samples. **(F)** UMAP of scRNA-seq data following annotation for normal tissue and normal organoids. **(G)** UMAP showing cell distribution in normal tissue and normal organoids; left panel: normal tissue, right panel: normal organoids. **(H)** Volcano plot of differential gene expression analysis for epithelial cells between normal tissue and normal organoids. **(I)** Enrichment analysis of top marker genes in epithelial cells from normal tissue and normal organoids; numbers within circles represent the count of differentially expressed genes.

We first performed a combined analysis of scRNA-seq data from normal tissue, normal organoids, KP organoids, KPP+ organoids, and organoid-derived tumor samples. For quality control, we filtered out cells expressing fewer than 100 genes and genes detected in fewer than 3 cells. Low-quality cells were filtered using the MAD method, and doublets were removed. Data integration was performed using scVI based on sample batch. A total of 79,684 cells were included in the analysis. Cell clustering was performed with a resolution of 1 (**Figure 2A**), resulting in 26 subpopulations. These subpopulations were annotated based on their specific marker genes. For example, EpCAM was used to identify epithelial cells; *Lum* and *Dcn* for fibroblasts; C1qa and Lyst1 for myeloid cells; Lyve1 and Prox1 for endothelial cells; *Cd3* for T cells; and *Acta2* and *Rgs5* for mural cells (**Figures 2B, C**). GSVA enrichment analysis showed that tumor samples exhibited higher activation of the KRAS signaling pathway, myogenesis pathway, and PI3K-AKT-mTOR signaling pathway (**Figure 2D**). We then compared normal tissue and normal organoid samples (**Figures 2F, G**). Differential gene expression analysis showed that in normal organoids, genes such as *Ly6a*, *Fgfbp1*, and *Agr2* were upregulated compared to normal mouse bladder tissue (**Figure 2H**). The activity of these genes is crucial for cell survival and regeneration, which aligns with the fact that organoids represent a process of cell regeneration and growth (23–25). Top marker gene enrichment analysis revealed that in normal tissue, pathways were enriched in stem cell pluripotency, unsaturated fatty acid synthesis, and fatty acid degradation, while in normal organoids, pathways were enriched in steroid biosynthesis and RNA splicing (**Figure 2I**).

### KPP+ tumor organoids display a distinct molecular profile relative to their KP counterparts

**Figure 3:**
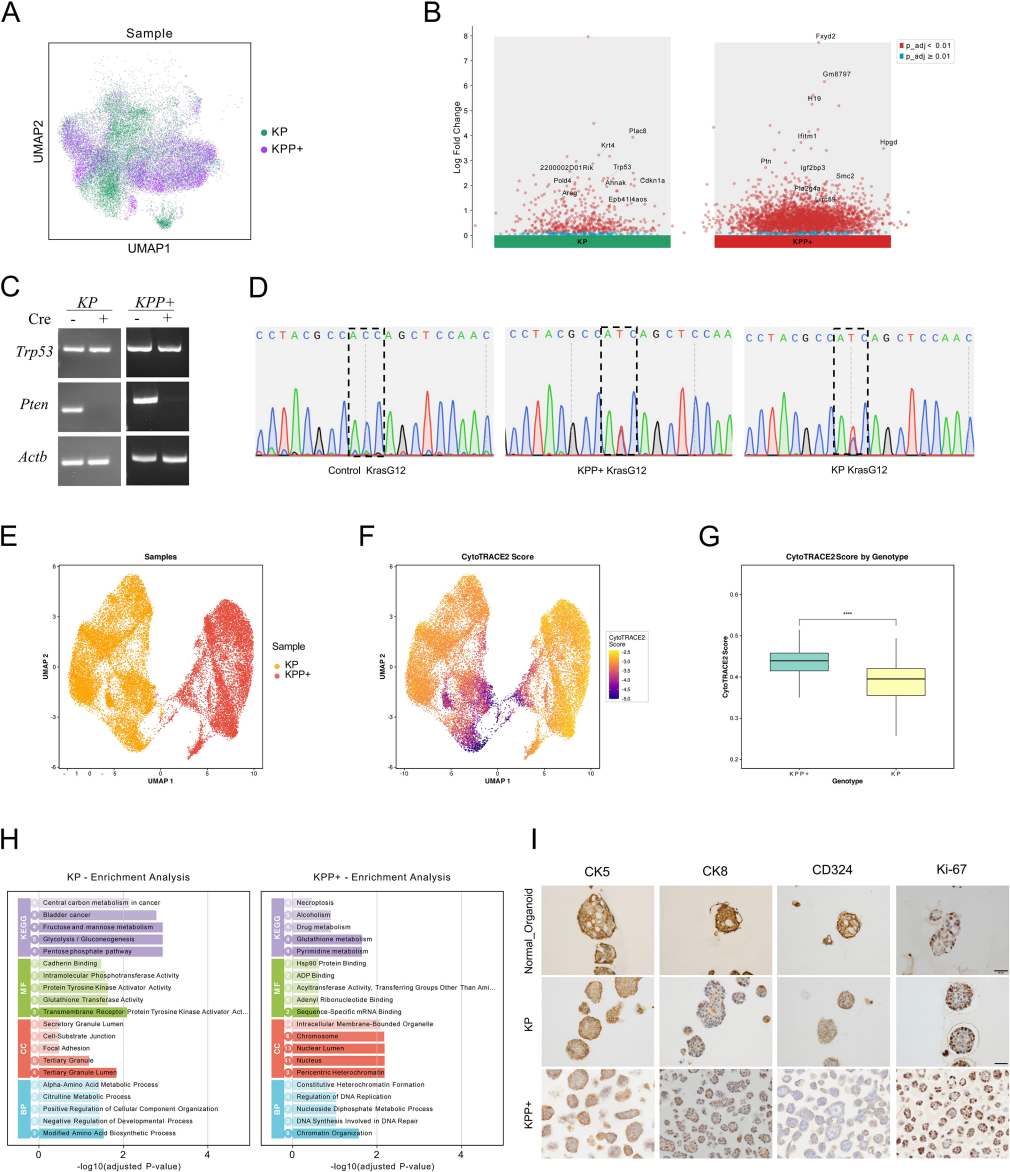
Characteristics of organoid culture and scRNA-seq analysis. **(A)** UMAP plot of epithelial cells in KP and KPP+ samples. **(B)** Volcano plot of differentially expressed genes in epithelial cells between KP and KPP+ samples. **(C)** Genomic PCR analysis of *Pten* and *Trp53* genotypes in KP and KPP+ cells. **(D)** Sanger sequencing validation of *Kras* mutations in KP and KPP+ samples. **(E)** CytoTRACE cell stemness analysis of epithelial cells in KP and KPP+ samples. **(F)** UMAP plot of CytoTRACE scores. **(G)** Box plots of CytoTRACE scores in epithelial cells from KP and KPP+ samples. **(H)** Enrichment analysis of differentially expressed genes in epithelial cells between KP and KPP+ samples. **(I)** IHC staining of different markers in normal bladder epithelial organoid, KP organoid, and KPP+ organoid. Scale bars: 20 μm.

To investigate the mechanism underlying the enhanced tumorigenicity of KPP+ organoids relative to KP. We compared the differences between epithelial cells in organoids of KP and KPP+ samples (**Figure 3A**). Differential gene expression analysis and analysis of top marker genes showed that *Krt4*, *Trp53*, and *Plac8* were significantly differentially expressed in KP samples, while Fxyd2 and Ptn were upregulated in KPP+ samples (**Figure 3B**). The genomic PCR analysis for genotyping KP and KPP+ cells confirmed the knockout status of *Pten* and *Trp53*. Gel electrophoresis results showed complete knockout of *Pten* in both KP and KPP+ cells. For *Trp53* in KPP+ cells, a heterozygous deletion was observed, indicated by a band intensity weaker than that of the control group (**Figure 3C**). Using cDNA from organoids, we performed PCR amplification of Kras exon 1 followed by Sanger sequencing. The results confirmed the presence of correct nucleotide mutations at the G12 position of Kras in both KP and KPP+ samples(**Figure 3D**). Pathway enrichment analysis indicated that KP samples were mainly enriched in metabolic pathways, while KPP+ samples were mainly enriched in drug metabolism and DNA repair pathways (**Figure 3G**). We also calculated the cell stemness scores of epithelial cells using CytoTRACE (**Figures 3E, F**). In KP samples, more cells had lower stemness scores (indicating lower stemness), while the overall stemness score was higher in KPP+ samples, suggesting that KPP+ samples exhibited higher stemness (**Figures 3G, H**). Immunohistochemical analysis revealed that both KP and KPP+ cells expressed the epithelial markers CK5, CK8, CD324 and Ki-67(**Figure 3I**).

### Organoid-derived tumors demonstrate superior modeling of human bladder cancer by more faithfully capturing its single-cell molecular and cellular communication profiles compared to the BBN-induced model

**Figure 4:**
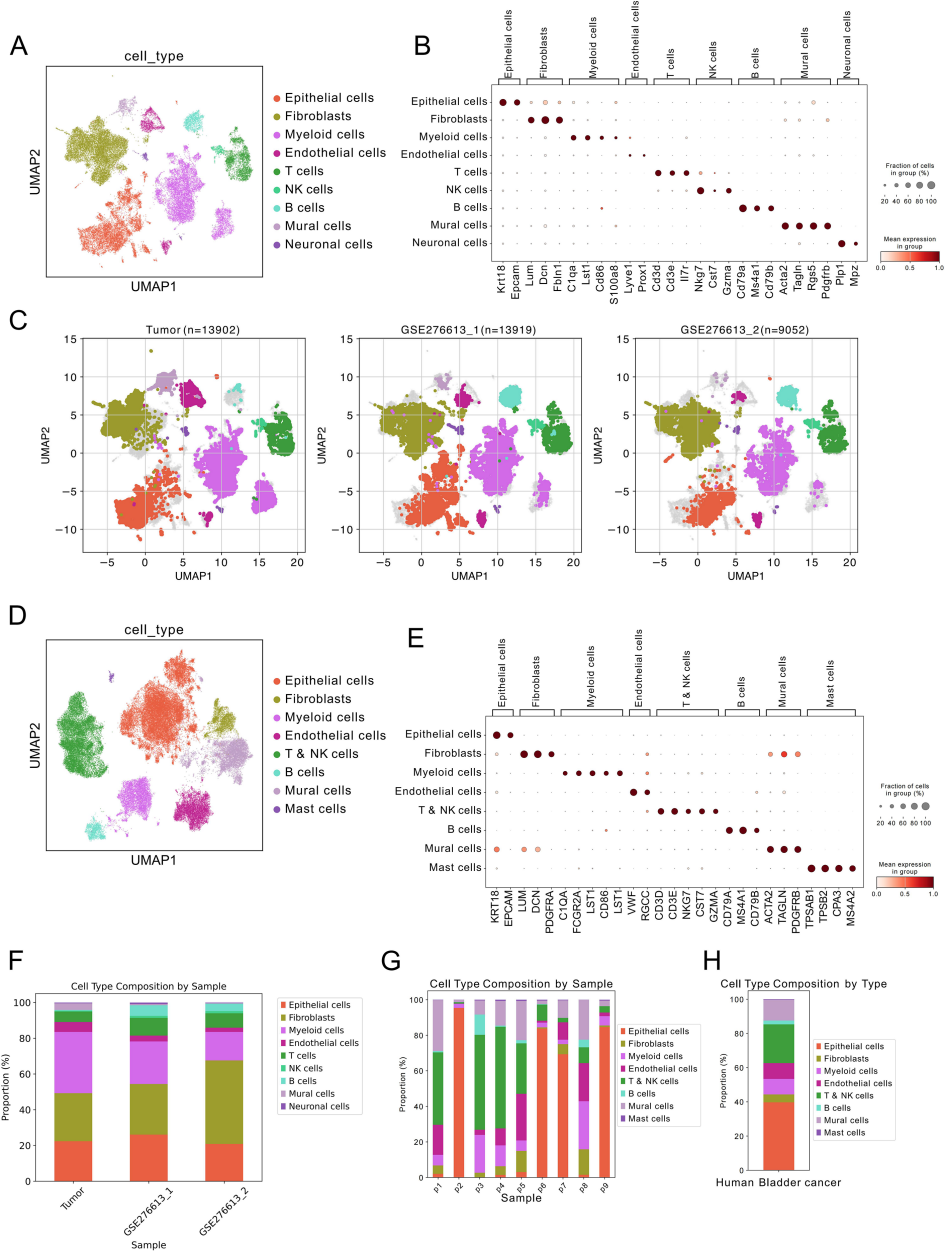
Comparison of scRNA-seq data from organoid-derived tumors, other mouse bladder cancer models, and human bladder cancer. **(A)** UMAP plot of integrated and annotated scRNA-seq data from organoid-derived tumors and BBN-induced mouse bladder cancer. **(B)** Dot plot showing marker genes for annotated cell subpopulations. **(C)** UMAP plot of cell classification in organoid-derived tumors and BBN-induced mouse bladder cancer. **(D)** UMAP plot of annotated scRNA-seq data from human bladder cancer. **(E)** Dot plot showing marker genes for cell subpopulations in human bladder cancer scRNA-seq data. **(F)** Proportion of different cell subpopulations across various samples from organoid-derived tumors and BBN-induced mouse bladder cancer. **(G)** Proportion of different cell subpopulations across various samples from human bladder cancer. **(H)** Proportion of different cell subpopulations in pooled samples from human bladder cancer.

Next, we compared the single-cell RNA sequencing data from our organoid-derived tumors, BBN-induced mouse bladder cancer, and human bladder cancer samples. We first integrated our tumor samples and BBN-induced mouse bladder cancer samples and annotated them according to previous rules (**Figures 4A, B**). Notably, B cells were almost absent in our tumor samples, while they were abundant in the BBN-induced mouse bladder cancer model (**Figure 4C**). We separately annotated the human bladder cancer samples, following the same general principles (**Figures 4D, E**). BBN-induced samples showed abundant B cells compared to our tumor samples. In the BBN-induced mouse bladder cancer samples, mural cells and myeloid cells were relatively reduced (**Figure 4F**). In human samples, significant heterogeneity was observed, with varying proportions of different cell types across different patient samples. In the pooled analysis, human samples showed fewer fibroblasts and lower proportions of B cells, but a relatively higher proportion of endothelial cells (**Figures 4G, H**).

**Figure 5:**
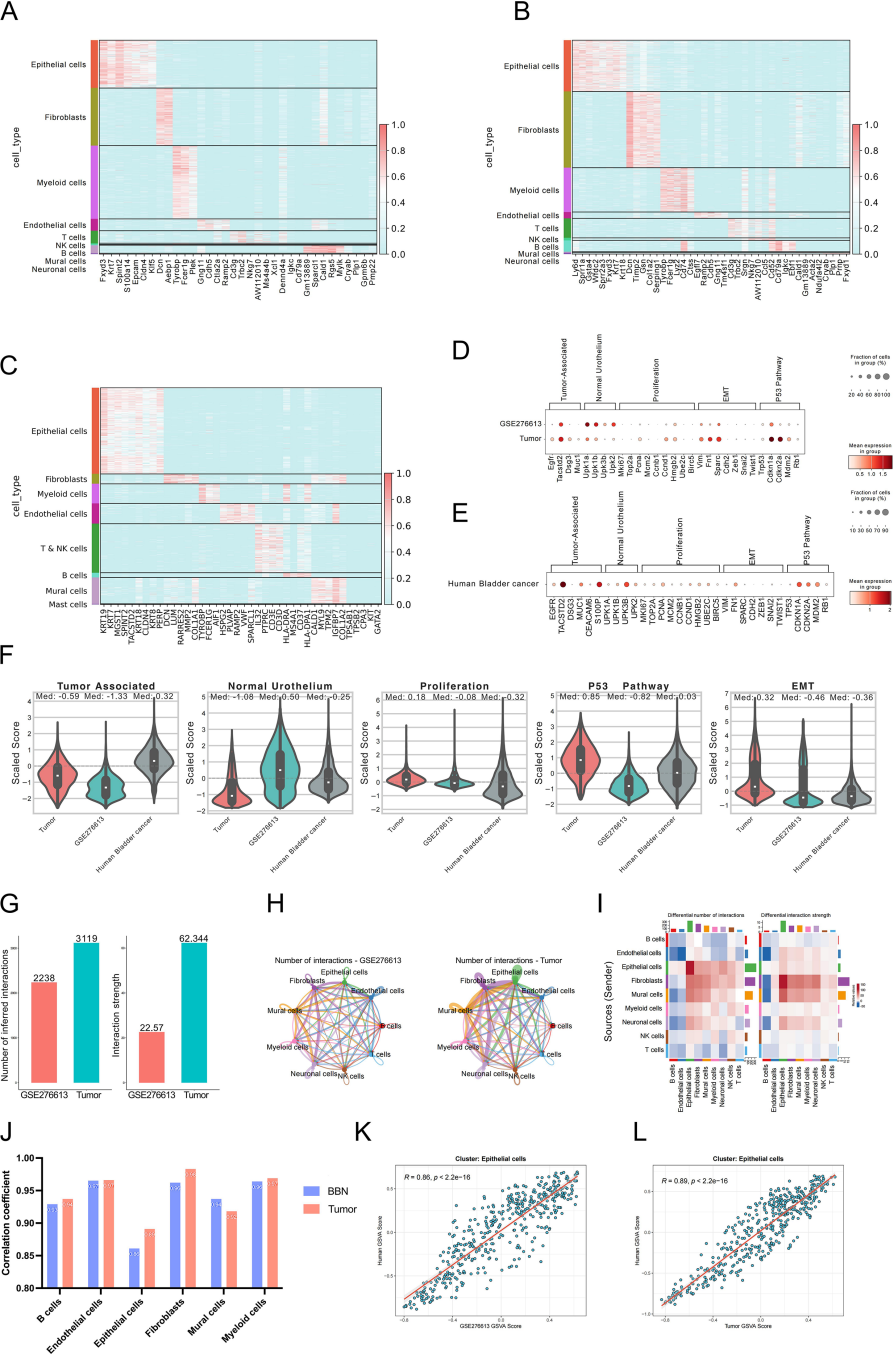
Transcriptomic landscape and cell-cell interaction analysis. **(A–C)** Heatmaps of top marker genes for different cell subpopulations in organoid-derived tumors, BBN-induced mouse bladder cancer, and human bladder cancer samples. **(D, E)** Dot plots (unscaled) showing the expression levels of different classification genes in epithelial cells from organoid-derived tumors and BBN-induced mouse bladder cancer **(D)** and human bladder cancer samples **(E)**. **(F)** Box plots of gene set activity scores for different gene sets in scRNA-seq epithelial cells, including tumor-related gene sets, normal bladder epithelium, proliferation, p53 pathway, and EMT gene sets. **(G)** Cell communication analysis of organoid-derived tumors and BBN-induced mouse bladder cancer, comparing the number and strength of cell-cell interactions. **(H)** Network diagram of cell-cell interactions. **(I)** Heatmap of the strength of cell-cell interactions between different cell subpopulations. **(J)** Bar chart showing the correlation of Bulk RNA gene set scores between scRNA-seq subsets in KPP and BBN-induced bladder cancer models and scRNA-seq subsets in human samples. **(K)** Correlation plot of Bulk RNA gene set score between scRNA-seq epithelial cell subsets in the BBN-induced bladder cancer model and scRNA-seq epithelial cell subsets of human samples. **(L)** Correlation plot of Bulk RNA gene set score between scRNA-seq epithelial cell subsets in the KPP bladder cancer model and scRNA-seq epithelial cell subsets of human samples.

We compared the top marker genes of different cell subpopulations across the three types of samples. In our tumor samples, epithelial cell-specific genes included *Fxyd3*, *Krt7*, *S100a14*, *Epcam*, and *Cldn4* (**Figure 5A**). In BBN-induced mouse bladder cancer, epithelial cell-specific markers were *Ly6d*, *Sprr1a*, *Gsta4*, *Fxyd3*, *Krt7*, and *Krt18* (**Figure 5B**). In human single-cell data, epithelial cell-specific markers were *KRT9*, *KRT7*, *MGST1*, *KRT18*, and *KRT8* (**Figure 5C**). Subsequently, we compared the expression of tumor-related genes (*EGFR*, *TACSTD2*, *DSG3*, *MUC1*, etc.), normal bladder epithelial markers (UPK family), and genes related to proliferation, EMT, and the p53 pathway in epithelial cells from the three different sample types. We observed that in BBN-induced mouse bladder cancer samples, the expression of normal bladder epithelial marker genes (UPK family) was higher, while in our samples and human bladder cancer samples, the expression of the tumor-related gene *TACSTD2* was higher. Our samples also showed higher expression of EMT-related genes (*VIM*, *FN1*, and *SPARC*) and, similar to human bladder cancer samples, higher expression of p53 pathway-related genes (**Figures 5D, E**).

The gene set scores showed that the tumor-related gene set score was highest in human samples, followed by our tumor samples and BBN-induced mouse bladder cancer samples. For the normal bladder tissue gene set, the BBN-induced mouse bladder cancer samples had the highest score. For the proliferation-related gene set, our tumor samples had the highest score. For the p53 pathway, our samples and human bladder cancer samples had higher gene set scores, and our samples also had the highest score for the EMT pathway gene set (**Figure 5F**). Comparing cell-cell communication between our tumor samples and BBN-induced mouse bladder cancer samples, our tumor samples showed more extensive cell-cell communication, both in terms of the number of interactions and the strength of communication, compared to the BBN-induced mouse bladder cancer samples (**Figures 5G–I**). Epithelial cells in KPP+ tumors showed a stronger correlation with human bladder cancer tissues in their transcriptomic signatures than did those from BBN-induced tumors(**Figures 5J–L**).

### The oncogenic transformation process in organoids may partially recapitulate the pathway of cancer development

**Figure 6:**
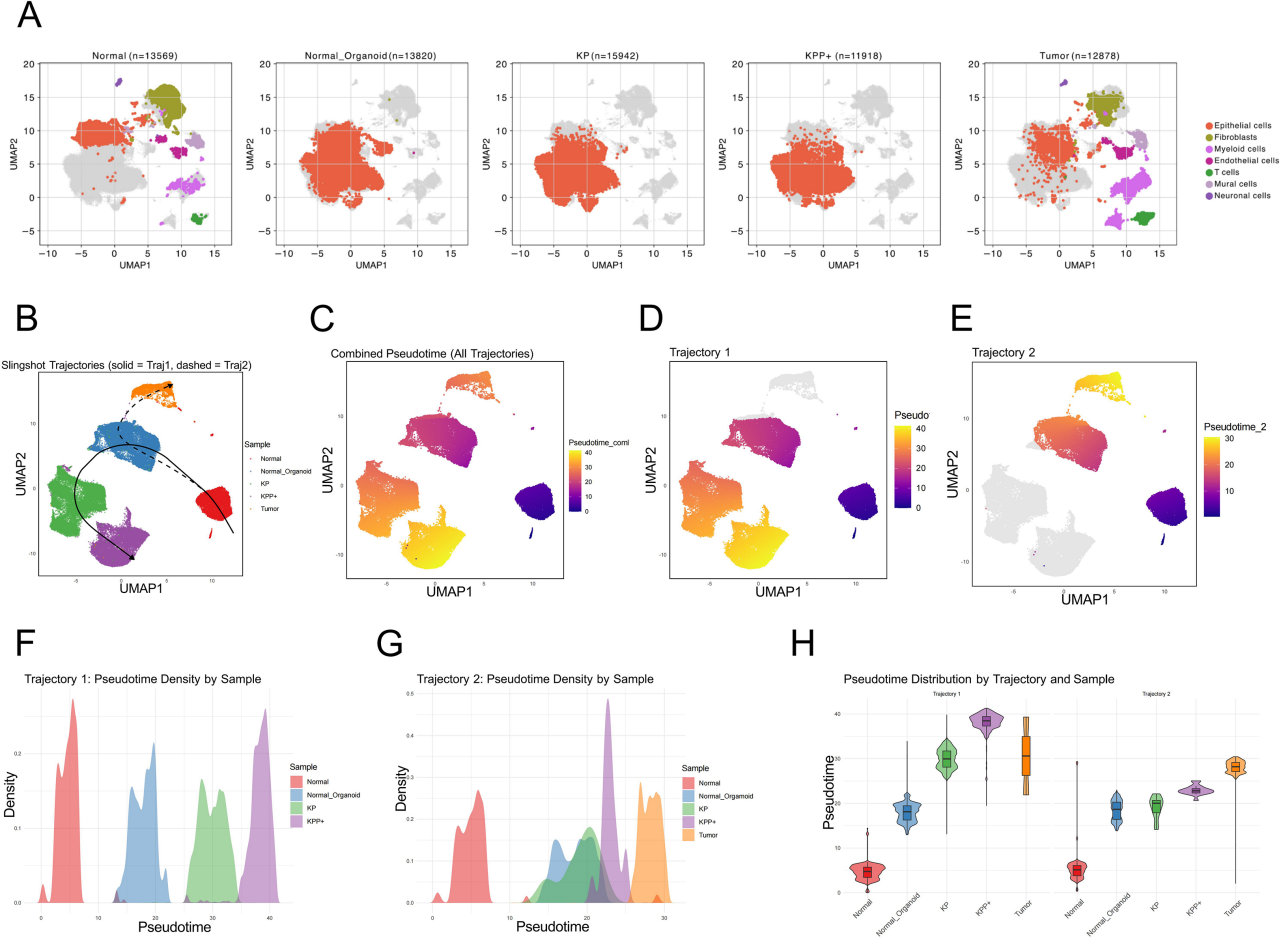
Slingshot pseudotime analysis of all models. **(A)** Faceted UMAP of all annotated samples. **(B)** All pseudotime trajectories generated by Slingshot analysis. **(C)** Pseudotime UMAP of the Slingshot analysis. **(D)** and **(E)** UMAP plots of trajectory 1 and trajectory 2 generated by Slingshot analysis. **(F)** and **(G)** Pseudotime distribution of samples involved in constructing the two pseudotime trajectories. **(H)** Violin plot comparing the pseudotime distribution of all samples across the two pseudotime trajectories.

Finally, we pooled all samples and extracted epithelial cells for Slingshot pseudotime analysis (**Figure 6A**). In the analysis, we designated normal mouse bladder tissue as the starting point but did not specify an endpoint. Our analysis ultimately obtained two pseudotime trajectories (**Figures 6B, C**). Trajectory 1 starts from normal tissue, progresses to normal organoid samples, then to KP samples, and finally to KPP+ samples (**Figures 6D, F**). Trajectory 2 starts from normal tissue, progresses to normal organoid samples, then to KP samples, and finally to tumor tissue (**Figures 6E, G**). In trajectory 1, the KPP+ samples had the highest pseudotime score, while in trajectory 2, the tumor samples had the highest pseudotime score (**Figure 6H**). Tumor tissues inherently represent a state that has evolved after overcoming immune surveillance, thus they cannot be directly compared to the *in vitro* organoid state. In contrast, at the organoid level, the normal tissue, normal organoids, KP, and KPP+ models form a clear developmental trajectory. This continuum provides novel insights into the progression of bladder cancer.

### Tumors derived from KPP+ organoids can serve as a platform for drug screening in bladder cancer

**Figure 7:**
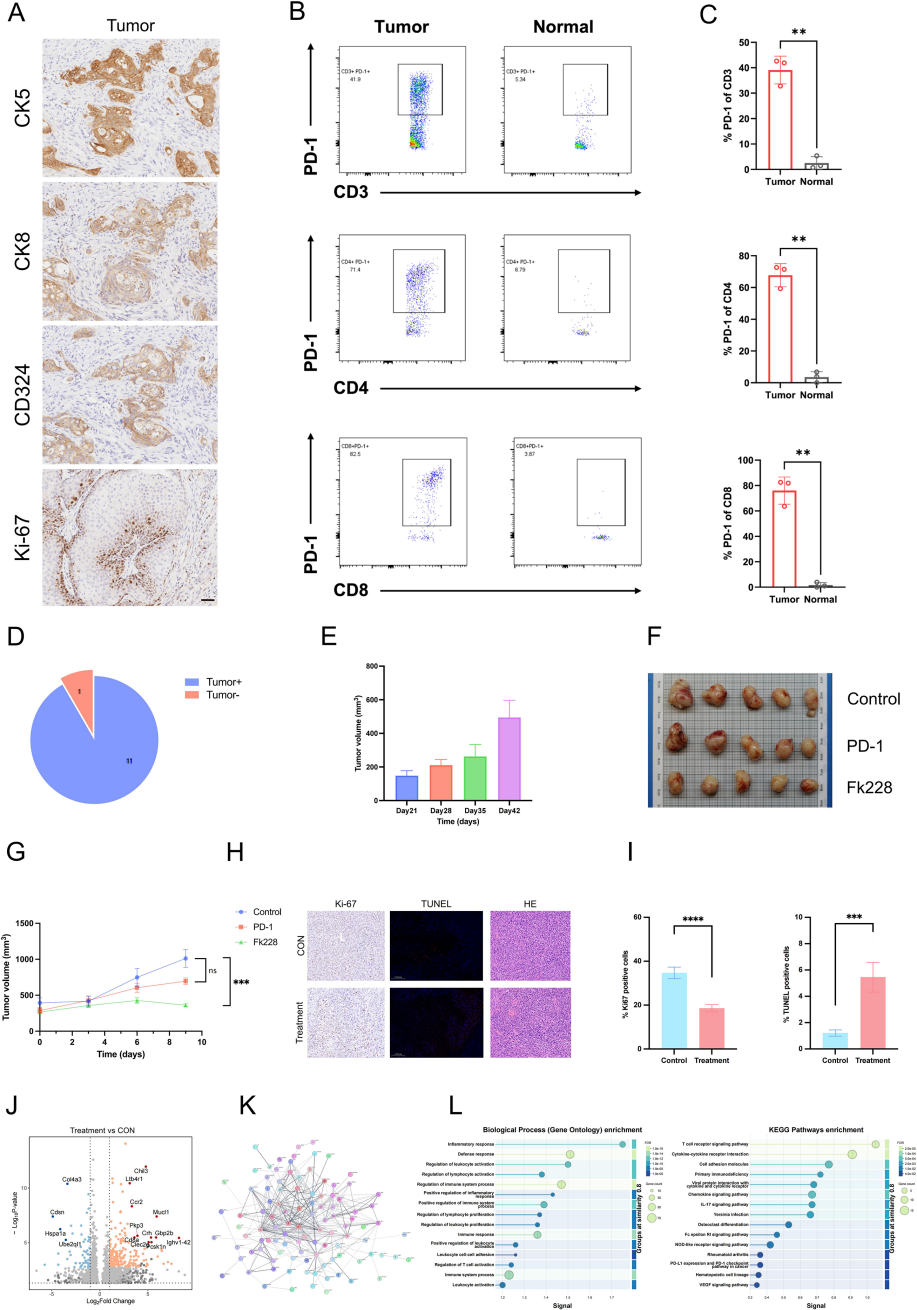
*In vivo* validation and therapeutic efficacy analysis. **(A)** IHC staining of different markers in KPP+ tumor tissue. Scale bar: 50 μm. **(B)** Representative flow cytometry plots showing PD-1 expression on CD3+ T cells, CD4+ helper T cells, and CD8+ cytotoxic T cells in tumor tissues compared to normal tissues. The gated regions indicate PD-1 positive populations, and the numbers represent the percentage of cells within the gate. **(C)** Quantification of the frequency of PD-1+ cells among CD3+, CD4+, and CD8+ T cell subsets in tumor and normal groups. (Data are presented as mean ± SEM; statistical significance was determined by Student’s t-test. **P < 0.01). **(D)** Tumor formation efficiency. Mice were subcutaneously inoculated bilaterally (on both flanks). The pie chart shows the incidence of tumor formation out of 12 injection sites (n=6 mice, 2 sites per mouse). “Tumor+” indicates successful tumor engraftment (n=11), and “Tumor-” indicates no tumor formation (n=1). **(E)** Tumor growth kinetics. Tumor volumes were monitored from Day 21 to Day 42 after inoculation (n=11). **(F)** Representative photographs of dissected tumors from mice in the Control, anti-PD-1 (PD-1), and FK228 treatment groups at the experimental endpoint (n=5 per group). **(G)** Tumor growth curves showing the changes in tumor volume (mm³) over time. Tumor volumes were measured every 3 days. (Data are presented as mean ± SEM. Statistical significance was determined using two-way ANOVA (or mixed-effects analysis) followed by Dunnett’s multiple comparisons test. ***P < 0.001; ns, not significant). **(H, I)** Ki67 IHC staining, TUNEL staining and HE of control and treatment group. (Data are presented as mean ± SEM; statistical significance was determined by Student’s t-test. ***P < 0.001, ****P < 0.0001; ns, not significant). **(J)** Volcano plot of differentially expressed genes in the treatment group compared to the control. **(K)** Protein-protein interaction (PPI) network of differentially expressed genes. **(L)** Pathway enrichment analysis of upregulated genes in the treatment group.

KPP+ organoid-derived tumors form subcutaneous masses in immunocompetent mice and maintain expression of the bladder cancer epithelial markers CK5, CK8, and CD324(**Figure 7A**). Additionally, KPP+ demonstrated a high tumor formation efficiency when subcutaneously inoculated into wild-type mice. Specifically, tumors developed in 11 out of 12 mice in 42 days (**Figures 7D, E**). To detect whether immune exhaustion occurs in our KPP+ tumors, we examined the PD-1 expression on CD3+, CD8+, and CD4+ T cells within the KPP+ tumors. The experimental results demonstrated that PD-1 expression on CD3+, CD8+, and CD4+ T cells all showed statistically significant high expression compared to the control group (**Figures 7B, C**). These findings indicate the presence of exhausted immune cells in our model. Given the biological fidelity of the KPP+ model, we assessed its potential as a drug screening platform for bladder cancer therapeutics. A comprehensive drug screening approach was implemented using a compound library including 328 epigenetic modulators, and the inhibitory efficacy and the relevant target pathways are shown in **Supplementary Figure S2**. Given its prominent presence in preclinical oncology literature among the top five performing agents, FK228 was consequently chosen as a lead candidate for follow-up studies. Consistent with our expectations, FK228 potently suppressed the growth of tumors derived from KPP+ organoids *in vivo*. The anti-tumor efficacy of FK228 surpassed that of the anti-PD-1 antibody used as a positive control(Fk228 vs. Control, *p = 0.0006*; PD-L1 vs. Control, *p = 0.0815*), suggesting that FK228 may be a potential therapeutic agent for bladder cancer (**Figures 7F, G**). To investigate the potential mechanisms by which FK228 inhibits bladder cancer growth, we performed Ki67 and TUNEL staining on tissues from both groups (**Figures 7H, I**). The results revealed that the percentage of Ki67-positive cells was significantly lower in the treatment group compared to the control group (p < 0.0001). In contrast, the proportion of TUNEL-positive cells was markedly higher in the treatment group than in the control group (p = 0.0010), indicating a greater extent of tumor cell apoptosis in the treatment group. At the same time, we performed bulk RNA sequencing and analysis on the final tumor tissue samples. The differential gene expression analysis revealed that genes such as *Cd8a*, *Pkp3*, *Ccr2*, and *Ltb4r1* were upregulated in the treatment group (**Figure 7J**). The protein-protein interaction (PPI) network of differentially expressed genes showed that genes such as *Tfrc*, *S100a8*, *Mmp8*, *Cd3d*, and *Ly6a* were located at the network center (**Figure 7K**). Pathway enrichment analysis of the differentially expressed genes indicated that the upregulated genes in the treatment group were primarily enriched in immune activation-related pathways, including inflammatory response, lymphocyte regulation, and T cell activation (**Figure 7L**).

## Discussion

Here, we established for the first time a systematic model of bladder tumorigenesis by combining GEMM-derived organoids with single-cell RNA sequencing. This platform allowed us to perform single-cell characterization of each distinct stage: normal tissue, normal organoids, oncogenically transformed organoids, and *in vivo* tumors. This comprehensive analysis of the entire tumorigenic process has not been previously achieved. In studies involving bladder cancer patients, it is exceptionally challenging to capture the early events of tumor initiation (26). Therefore, utilizing organoid models to study bladder cancer development represents a highly effective strategy (27, 28). A significant hurdle with using normal human bladder organoids is the considerable difficulty in introducing multi-site edits and precise mutations via gene editing. The low efficiency of this process typically leads to substantial cell loss, which is not conducive to understanding cancer initiation. In contrast, organoids derived from genetically engineered mice do not face this limitation. Lentiviral vectors carrying Cre recombinase enable highly efficient infection and genetic recombination in these organoids (29). This method better preserves cellular heterogeneity throughout the process. Furthermore, the transformed cancerous organoids can be re-transplanted subcutaneously into immunocompetent mice to establish tumor models capable of immune evasion (30, 31). Consequently, this approach currently stands as a highly effective model for investigating the initiation and progression of bladder cancer.

It is noteworthy that throughout this process, GEMM-derived organoids of different genotypes exhibited distinct cellular characteristics and fates. Although the KPP+ model differs from the KP model only by the loss of one allele of *Trp53*, the KPP+ organoids demonstrated enhanced stem cell-like properties and the capability to form subcutaneous tumors in immunocompetent mice. This observation provides critical insights into cancer progression and immune evasion. Given that *TP53* is frequently mutated in bladder cancer and plays a pivotal role in disease advancement, our model effectively recapitulates this key aspect (32, 33). In subsequent studies, we will explore whether organoids derived from mice with complete *Trp53* knockout retain tumor-forming ability. These findings underscore the importance of considering the critical function of Trp53 in the development of future models.

The clinical relevance of our tumor model represents the most critical aspect of this system. In our study, KPP+ organoids formed subcutaneous tumors, whose molecular characteristics were comprehensively profiled using single-cell RNA sequencing. These profiles were then compared with data from both BBN-induced mouse models and human bladder cancer patients. Our KPP+-derived tumor model exhibited closer resemblance to human tumors than the BBN-induced model, particularly in the recapitulation of patient-specific pathways such as the p53 and EMT signaling pathways. These analyses provide clear evidence supporting the clinical relevance of our model. The BBN-induced model faces significant challenges in tumor feature stability and reproducibility due to its undefined genetic mutation profile (34). In contrast, our GEMM-derived tumor model, with well-defined genetic deletions and mutations, offers superior stability and is therefore more suitable for investigating bladder cancer with specific molecular characteristics.

A key innovation of our work lies in utilizing the oncogenic transformation process of organoids to study cancer initiation. Our Slingshot pseudotime analysis successfully reconstructed two distinct cellular trajectories. One trajectory, progressing from normal tissue to normal organoids, KP organoids, and finally KPP+ organoids, largely aligns with the expected path of cancer development. However, it is noteworthy that tumors derived from KPP+ organoids did not occupy the endpoint of this trajectory, which we consider a limitation of the current analysis. A likely explanation is that KPP+ derived tumors result from a secondary tumor formation process, during which cellular characteristics undergo significant changes, posing considerable challenges for trajectory inference. To address this, we plan to employ barcoding and cell lineage tracing systems in future studies to obtain more definitive evidence. In the second trajectory, KP organoids progressed towards tumor tissue, an observation inconsistent with our experimental findings and supported by a low reliability assessment. This discrepancy, in turn, reinforces the validity of the primary trajectory involving the KPP+ organoids. While our pseudotime analysis provides valuable insights into the transcriptomic heterogeneity of KPP+ tumors, we acknowledge several limitations inherent to this computational approach. First, trajectory inference algorithms reconstruct potential developmental paths based on transcriptomic similarity, which does not necessarily equate to chronological evolutionary history. Specifically, inferring a continuous trajectory from normal urothelial cells to established tumor populations implies a linear progression that may oversimplify the complex, branching evolution of cancer. The distinct biological contexts of normal tissue versus the tumor microenvironment can introduce batch effects or biological disconnects that pseudotime algorithms might misinterpret as transitional states.

Therefore, the trajectories presented in this study should be interpreted as a reflection of transcriptomic continuity and cellular plasticity within the tumor ecosystem, rather than a definitive reconstruction of the tumor’s evolutionary origin. Our findings highlight the existence of intermediate cell states and potential gene expression gradients that contribute to intratumoral heterogeneity. These computational predictions serve as hypothesis-generating frameworks, suggesting potential regulatory mechanisms that drive phenotypic shifts, which warrant further validation through lineage-tracing experiments or time-series sampling.

Another important application of our tumor model is its capability for drug sensitivity evaluation. Our KPP+ model demonstrated robust performance in both *in vitro* and *in vivo* drug assessments. Specifically, we identified FK228 as a hit compound during drug screening and validated its efficacy *in vivo*. RNA-seq analysis revealed the activation of signaling pathways related to inflammatory response, lymphocyte regulation, and T cell activation following FK228 treatment. These results suggest that FK228, as a small-molecule HDAC inhibitor, not only suppresses tumor growth but may also modulate the interaction between tumor cells and the immune microenvironment to collectively inhibit tumor progression (35, 36). This mechanism likely would not have been observed in immunodeficient tumor models, thereby providing stronger support for the physiological relevance and effectiveness of our model system.

Recently, patient-derived organoids (PDOs) are extensively cultured for research on individualized variations of tumors and drug screening. Plenty of studies have demonstrate the superiority of PDOs as *in vitro* models for tumors. Shen’s group generated a biobank of 22 patient-derived organoid lines that recapitulates the histopathological and molecular diversity of human bladder cancer. The organoid lines often retain parental tumor heterogeneity, which represent a model system for treatment response (37). Teoh’s group established a bladder cancer PDOs biobank with 36 organoids from 56 patients. They conducted drug screening tests to identify differential drug sensitivities among the PDOs, which was consistent with PDO xenograft animal studies and patients’ clinical treatment outcomes (38). PDOs can replicate the histological and genomic features of parental tumors, making them excellent models for precise and personalized treatment strategies (39). However, they still have some limitations. Limited passage times and low survival rate restrict organoid lines from being used for long-term studies, and the inconvenience of obtaining tumor tissue increases the uncertainty in organoids generation (40). Another limitation of PDOs lies in the fact that *in vivo* experiments with PDOs rely on immunodeficient mice, which prevents the exploration of the influence of immune microenvironment on tumor evolution. In contrast, the mouse-derived organoids established in our study could avoid these issues. Relying on GEMMs, organoids in this study possess stable genotypes and highly efficient tumorigenic conversion rates, which is suitable for generalized and universal long-term tumor progression studies. Additionally, organoids derived from mice can be engrafted into immunocompetent hosts, thereby enabling a more accurate modeling of the practical tumor microenvironment. This is highly favorable for investigating the effects of the immune system on tumor progression. Certainly, our mouse-derived organoids also have shortcomings. Since mice and humans exhibit species diversity, organoids derived from mice are not capable of expressing particular mutations and therapeutic targets specific to human bladder cancer, as well as the heterogeneity of cellular phenotypes. This limits its application to studies concerning specific mutations and the development of personalized, precision therapies for specific therapeutic targets. A feasible alternative is to combine patient-derived organoids with GEMMs models. Through deep sequencing, the mutation profiles of patient-derived organoids can be characterized to generate corresponding GEMMs. Bladder tissue extracted from these mice can be used to establish bladder cancer organoids *in vitro* for drug screening. Additionally, these organoids can be engrafted into immunocompetent mice to investigate tumor evolution *in vivo* and the effects of the immune microenvironment on tumor progression.

In summary, our model and methodology provide novel insights into both the pathogenesis and treatment of bladder cancer. This approach can also be broadly applied to the study of other cancer types. Tumors formed by GEMM-derived organoids through immune evasion can be utilized for evaluating drug sensitivity and efficacy, offering a critical platform for the future development and testing of immune-related therapeutics for bladder cancer, representing a highly valuable platform for studying the development, progression, immune evasion, and drug screening in bladder cancer.

## Methods and materials

### scRNA-seq analysis

Data analysis was primarily performed using Scanpy (Python) and Seurat (R programming language). For quality control, cells expressing fewer than 100 genes and genes detected in fewer than 3 cells were filtered out. Cells were filtered using the MAD method with an outlier threshold of 5; cells with mitochondrial gene expression rates (pct_counts_mt) greater than 20% or mitochondrial outlier scores (mt_outlier) greater than 5 were also filtered. Doublets were removed using sc.pp.scrublet. For data integration across different single-cell sequencing samples, scVI was used with max_epochs set to 400 and early_stopping_patience set to 15; the latent representation (X_scVI) was obtained using get_latent_representation. Clustering was performed using sc.tl.leiden with a resolution of 1.0. sc.tl.score_genes was used to calculate gene set scores. scRNA-seq data from a BBN-induced mouse bladder cancer model (GSE276613) and human bladder cancer samples (GSE222315) were obtained from the GEO database.

### Bulk RNA-seq analysis

The gene expression matrix was obtained using FastQC and featureCounts, with the genome reference version GRCm39.113. Differential gene expression analysis was performed using the R package DESeq2. PPI network construction and enrichment analysis of differentially expressed genes were performed using STRING. When calculating the specificity score of single-cell RNA sequencing subpopulations in bulk RNA, the GSVA method was used to calculate the score of the TOP marker gene set for each single-cell subpopulation. Spearman correlation was used to calculate the correlation coefficient.

### CytoTRACE cell stemness scoring

CytoTRACE2 was used to calculate the stemness scores of epithelial cells. During calculation, cell counts were used with a batch size of 10,000 and a smoothing batch size of 1,000. The plotData function retrieved the calculated results and plotting information, including data such as CytoTRACE2_Boxplot_byPheno, Phenotype_UMAP, and CytoTRACE2_UMAP.

### Slingshot pseudotime analysis

Pseudotime analysis was performed using the Slingshot package. Only epithelial cells were used to construct the pseudotime trajectory. The “Normal” sample was designated as the starting cluster (start.clus), and end.clus was set to NULL. This yielded all pseudotime trajectories, which were then analyzed separately for pseudotime values and trajectory paths. Box plots displayed the distribution of pseudotime values, while density plots showed the order of pseudotime progression.

### CellChat intercellular communication analysis

The CellChat package was used for intercellular communication analysis. This analysis was performed separately on single-cell data from organoid-derived tumors and BBN-induced mouse bladder cancer. All signaling pathways were included in the analysis, and the results from the two datasets were integrated using mergeCellChat. The compareInteractions function was used to perform an overall comparison of cell-cell communication patterns.

### Organoid culture

Bladder tissues were harvested from mice and rinsed in PBS (BasalMedia). The tissues were then minced with surgical scissors and digested in a solution of 10% collagenase/hyaluronidase (STEMCELL Technologies) diluted in DMEM/F12 (Gibco) supplemented with 5% FBS. Subsequent digestion was performed sequentially using TrypLE (Thermo Fisher Scientific) and dispase (STEMCELL Technologies) supplemented with a 1:10 dilution of 1 mg mL−1 DNase I (STEMCELL Technologies). The resulting cell suspension was filtered through a 40-μm cell strainer. Cells were resuspended in Matrigel (Corning) and seeded as 50-μL droplets (50 μL per well) in a 24-well plate. The culture medium was supplemented with Cre-expressing lentivirus on day 7. Approximately ten days later, GFP-positive cells were selected based on GFP fluorescence and re-plated at a density of 2,000 cells per well. The organoids were passaged weekly.

### Organoid genotyping

After collecting the organoids, genomic DNA and total RNA were isolated using Beyotime’s DNA and RNA extraction kits, respectively. The RNA was then reverse-transcribed into complementary DNA (cDNA) using a reverse transcription kit.Genotype identification was performed as follows: For the Pten gene, a PCR assay was conducted using primers specific to its fifth exon (Pten-F: GCACAGTATCCTTTTGAAGACC; Pten-R: TTGTCTCTGGTCCTTACTTC).For the Trp53 gene, a PCR assay was conducted using primers specific to its third exon (Trp53-F:CCATCACCTCACTGCATGGA; Trp53-R:TGTCCCAGACTGCAGGAAGC). For the Kras gene, the cDNA was used as a template for PCR amplification targeting its first exon, followed by Sanger sequencing of the product.

### Flow cytometry

Following tumor dissection, tissues were finely minced and enzymatically digested in 1× Collagenase/Hyaluronidase solution (Stemcell, cat# 07912) supplemented with 1 U/mL Dispase II (Stemcell, cat# 07913) and 0.1 mg/mL DNase I (Stemcell, cat# 07962) at 37 °C for 1 hour with gentle agitation. The digested cell suspension was quenched with HBSS containing 2% fetal bovine serum (FBS), filtered through a 70 µm cell strainer, pelleted by centrifugation, and resuspended in PBS containing 1% bovine serum albumin (BSA). Cells were incubated with an anti-mouse CD16/32 antibody on ice for 15 minutes to block nonspecific Fc receptor binding, then stained with a cocktail of fluorochrome-conjugated antibodies for 30 minutes on ice in the dark. The antibody panel included: APC anti-mouse CD45 (BioLegend, cat# 103111), PE/Cyanine7 anti-mouse CD45 (BioLegend, cat# 147703), Alexa Fluor^®^ 700 anti-mouse CD3 (BioLegend, cat# 100215), FITC anti-mouse CD4 (BioLegend, cat# 100405), Pacific Blue™ anti-mouse CD8a (BioLegend, cat# 100728), PE anti-mouse CD279 (PD-1) (BioLegend, cat# 135205), Pacific Blue™ anti-mouse/human CD11b (BioLegend, cat# 101223), and APC anti-mouse CD274 (B7-H1, PD-L1) (BioLegend, cat# 124311). After two washes with PBS, cells were incubated with DAPI (1:2000 dilution) in FACS buffer for 10 minutes for live/dead discrimination. Samples were acquired on a BD LSR Fortessa flow cytometer (BD Biosciences), and data were analyzed using FlowJo software.

### Animal experiments

All animal experiments were conducted in the specific pathogen-free (SPF) facility of the Animal Center at Renji Hospital, Shanghai Jiao Tong University School of Medicine. All procedures were approved by the Institutional Animal Care and Use Committee of the hospital. Genetically edited mice were bred by Cyagen Biosciences and maintained under SPF conditions throughout breeding and experimentation.

All mice were euthanized by cervical dislocation, performed by professionally trained personnel from the Experimental Animal Center of Renji Hospital, School of Medicine, Shanghai Jiao Tong University. The procedure was carried out swiftly to ensure instantaneous death and minimize animal distress, in strict accordance with the AVMA (American Veterinary Medical Association) Guidelines for the Euthanasia of Animals.

To establish the tumor model, 1 × 10^6 cells were subcutaneously injected into one flank of each male C57BL/6 mouse. Mice were randomly assigned to treatment groups when tumors became palpable approximately two weeks post-inoculation. The mice in the treatment group received FK228 (1 mg/kg, TargetMol, Cat# T6006) via intraperitoneal injection twice weekly. Control mice received vehicle injections on the same schedule. Mice were euthanized before the tumor volume reached the ethically permitted limit. Tumors were then harvested, and their length and width were measured using a vernier caliper. Tumor volume was calculated using the formula: (Length × Width²)/2.

### Drug sensitivity assay

Organoids were dissociated into single cells, counted, and seeded in a 96-well plate at a density of 2,000 cells per well. The following day, compounds were added to a final concentration of 5 μM, and the cells were incubated for 72 hours. Cell viability was quantified using a CCK-8 assay kit (Life-iLab) according to the manufacturer’s protocol. A compound was considered for further selection if it induced a reduction in cell viability greater than 50% (i.e., inhibition ≥ 50%).

### Histology, immunohistochemistry, and TUNEL staining

Resected mouse tumor tissues were fixed in 10% formalin overnight, followed by dehydration and embedding in paraffin. Tumor sections (4-5 μm) were deparaffinized with xylene and rehydrated through a graded ethanol series (100%, 95%, and 70%). For antigen retrieval, the sections were heated in Improved Citrate Antigen Retrieval Solution (Beyotime). Endogenous peroxidases were blocked using an Enhanced Endogenous Peroxidase Blocking Buffer (Beyotime), and non-specific binding sites were blocked with 10% goat serum. The sections were then incubated with primary antibodies at 4 °C overnight. On the following day, the sections were incubated with HRP-conjugated secondary antibodies, and the signal was developed using a DAB Peroxidase Substrate Kit for IHC (Yellow Color) (Yeason). Cell nuclei were counterstained with hematoxylin.

The TUNEL assay was performed following the manufacturer’s protocol (YEASON, Cat# 40306ES20).

For the quantification of Ki-67 and TUNEL staining, five random fields per tissue section were selected. The percentage of positive cells was determined using ImageJ software.

### Statistical analysis

All statistical analyses and the generation of bar graphs were performed using GraphPad Prism software (version 10.1.2). The significance level was set at P<0.05.

## Data Availability

Publicly available datasets were analyzed in this study. This data can be found here: https://ngdc.cncb.ac.cn/omix/, accession number OMIX015645.

## Supplementary material

Supplementary Figure 1 Mutation Status of TP53, KRAS, and PTEN in Bladder Cancer Patients from the TCGA Database. **(A)** Summary of somatic mutation profiles in the TCGA bladder urothelial carcinoma (TCGA-BLCA) cohort. The upper panel shows the total number of somatic mutations per tumor sample, with outliers removed and the median mutation count indicated. The lower panel displays the distribution of variant classifications, variant types, and single nucleotide variants (SNVs) across all samples. **(B)** Co-mutation analysis of *TP53*, *KRAS*, and *PTEN* in the TCGA-BLCA cohort. Tumor samples were classified based on the presence or absence of mutations in these three genes, and the number of samples in each mutation combination is shown. **(C)** Oncoplot depicting the mutation landscape of the top 20 most frequently mutated genes in TCGA-BLCA samples. Each column represents a tumor sample and each row represents a gene. Colored blocks indicate different mutation types, while non-mutated samples are omitted for clarity. **(D)** Visualization of the mutation status of the *TP53*, *KRAS*, and *PTEN* genes in individual tumor samples, highlighting their mutation patterns and co-occurrence relationships. **(E)** Ti/Tv (transition/transversion) analysis of somatic single nucleotide variants in TCGA-BLCA samples, illustrating the relative contribution of different nucleotide substitution types. **(F)** Allele frequency plot of bladder cancer based on TCGA-BLCA data.

Supplementary Figure 2 Hierarchical Clustering and Heatmap Analysis of Small-Molecule Inhibitor Screening. This heatmap illustrates the inhibitory effects of a diverse library of small-molecule compounds, categorized by their primary biological pathways and molecular targets. Each column represents an individual compound, while the primary row displays the normalized Inhibition Percentage (Inhibition_pct). The color gradient represents the Z-score of the inhibition percentage, ranging from deep red (high inhibitory activity) to deep blue (low inhibitory activity). The top annotation bar classifies drugs into functional pathways (e.g., Apoptosis, Cell Cycle), and the second bar indicates specific molecular targets (e.g., HDAC, Topoisomerase). The dendrogram indicates unsupervised hierarchical clustering based on inhibitory profiles.
