# Supplementary figures and images for: Rapid establishment of KRAS-driven bladder cancer initiation and immune escape models using genetically engineered mice and organoid approaches
Source: Front Immunol. 2026 Mar 23;17:1726443. doi: 10.3389/fimmu.2026.1726443 (PMC13050906; doi:10.3389/fimmu.2026.1726443)

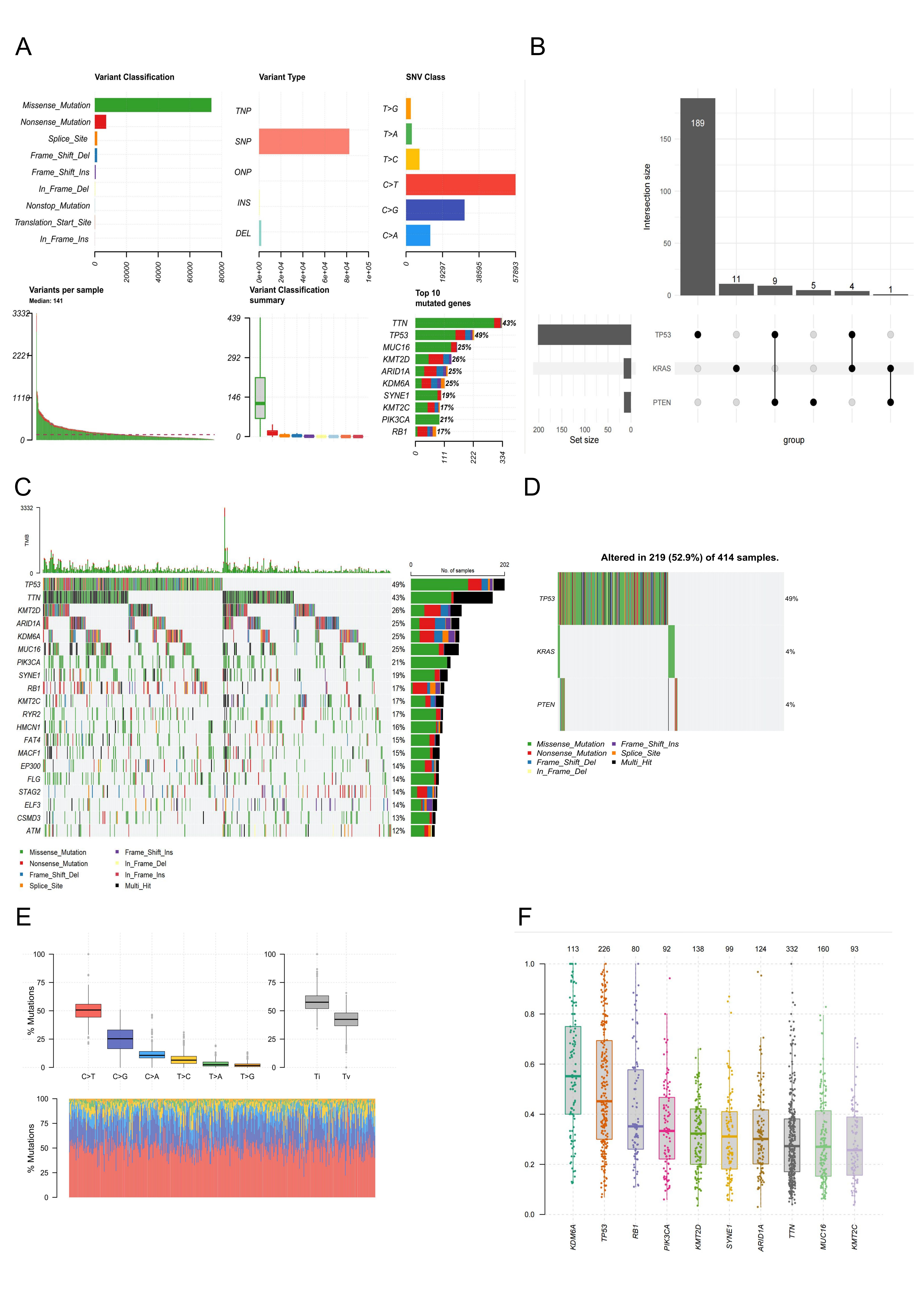

Supplement: Supplementary Figure 1 — Mutation Status of TP53, KRAS, and PTEN in Bladder Cancer Patients from the TCGA Database. (A) Summary of somatic mutation profiles in the TCGA bladder urothelial carcinoma (TCGA-BLCA) cohort. The upper panel shows the total number of somatic mutations per tumor sample, with outliers removed and the median mutation count indicated. The lower panel displays the distribution of variant classifications, variant types, and single nucleotide variants (SNVs) across all samples. (B) Co-mutation analysis of TP53, KRAS, and PTEN in the TCGA-BLCA cohort. Tumor samples were classified based on the presence or absence of mutations in these three genes, and the number of samples in each mutation combination is shown. (C) Oncoplot depicting the mutation landscape of the top 20 most frequently mutated genes in TCGA-BLCA samples. Each column represents a tumor sample and each row represents a gene. Colored blocks indicate different mutation types, while non-mutated samples are omitted for clarity. (D) Visualization of the mutation status of the TP53, KRAS, and PTEN genes in individual tumor samples, highlighting their mutation patterns and co-occurrence relationships. (E) Ti/Tv (transition/transversion) analysis of somatic single nucleotide variants in TCGA-BLCA samples, illustrating the relative contribution of different nucleotide substitution types. (F) Allele frequency plot of bladder cancer based on TCGA-BLCA data. [file Image1.jpeg]

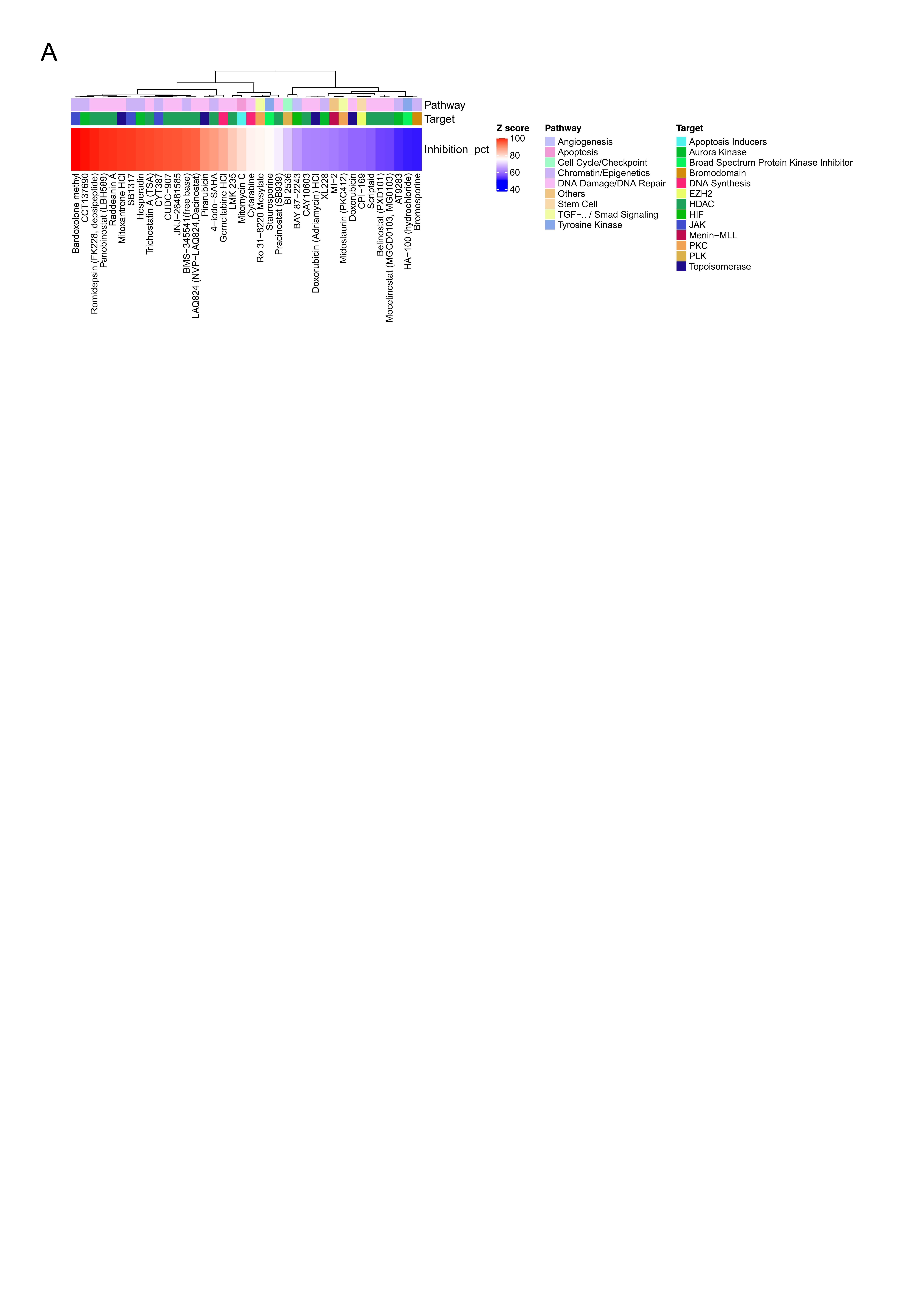

Supplement: Supplementary Figure 2 — Hierarchical Clustering and Heatmap Analysis of Small-Molecule Inhibitor Screening. This heatmap illustrates the inhibitory effects of a diverse library of small-molecule compounds, categorized by their primary biological pathways and molecular targets. Each column represents an individual compound, while the primary row displays the normalized Inhibition Percentage (Inhibition_pct). The color gradient represents the Z-score of the inhibition percentage, ranging from deep red (high inhibitory activity) to deep blue (low inhibitory activity). The top annotation bar classifies drugs into functional pathways (e.g., Apoptosis, Cell Cycle), and the second bar indicates specific molecular targets (e.g., HDAC, Topoisomerase). The dendrogram indicates unsupervised hierarchical clustering based on inhibitory profiles. [file Image2.jpeg]
